# Experimental characterization of four ionization chamber types in magnetic fields including intra-type variation

**DOI:** 10.1016/j.phro.2024.100561

**Published:** 2024-02-25

**Authors:** Stephan Frick, Moritz Schneider, Ralf-Peter Kapsch, Daniela Thorwarth

**Affiliations:** a Physikalisch-Technische Bundesanstalt, Braunschweig, Germany; b Section for Biomedical Physics, Department of Radiation Oncology, University Hospital Tübingen, Tübingen, Germany; c German Cancer Consortium (DKTK), Partner site Tübingen, A Partnership between DKFZ and University Hospital Tübingen, Germany

**Keywords:** MR guided radiation therapy, Magnetic field correction factor, MR-optimized ionization chamber, Dosimetry

## Abstract

**Background and purpose:**

For dosimetry in magnetic resonance (MR) guided radiotherapy, assessing the magnetic field correction factors of air-vented ionization chambers is crucial. Novel MR-optimized chambers reduce MR-imaging artefacts, enhancing their quality assurance utility. This study aimed to characterize two new MR-optimized ionization chambers with sensitive volumes of 0.07 and 0.016 cm^3^ regarding magnetic field correction factors and intra-type variation and compare them to their conventional counterparts.

**Material and methods:**

Five chambers of each type were evaluated in a water phantom, using a clinical linear accelerator and an electromagnet, as well as a 1.5 T MR-linac system. The magnetic field correction factor $k_{\vec{B},Q}$, addressing the change of response caused by a magnetic field, was assessed together with its intra-type variation. MR-optimized and conventional chambers were compared using a Mann-Whitney U-Test.

**Results:**

Considering 1.5 T and a perpendicular chamber orientation, we observed significant differences in the magnetic field-induced change in chamber reading between the two 0.016 cm^3^ chamber versions (p = 0.03). For a 7 MV beam, MR-optimized chambers (0.016/0.07 cm^3^) showed $k_{\vec{B},Q}$ values of 1.0426(66) and 1.0463(44), compared to 1.0319(53) and 1.0480(41) of their conventional counterparts. In anti-parallel orientation, $k_{\vec{B},Q}$ was 1.0012(69) and 0.9863(49) for the MR-optimized chambers. The average intra-type variation of $k_{\vec{B},Q}$ over all chamber types was 0.3%.

**Conclusion:**

Magnetic field correction factors were successfully determined for four ionization chamber types, including two new MR-optimized versions, allowing their use in MR-linac absolute dosimetry. Evaluation of the intra-type variation enabled the assessment of their contribution to the uncertainty of tabulated $k_{\vec{B},Q}$.

## 1 Introduction

The combination of real-time magnetic resonance (MR) imaging and a linear accelerator (linac) promises to improve the quality of treatment in radiation oncology [1]. Hybrid devices, so-called MR-linacs, allow for online adaptive radiotherapy in which a treatment plan is adjusted to account for daily changes in a patient’s anatomy as revealed by on-couch MR images [2]. Using continuous MR-imaging, gating and tracking of moving targets seems feasible in MR-guided radiotherapy [3].

With the increasing use of MR-linac systems in clinical routine, robust, fast and accurate dosimetry protocols are required for quality assurance. Recent studies have characterized air-vented ionization chambers regarding their behaviour in magnetic fields. The response of these chambers has been shown to change, as a function of an external magnetic field [4], [5], [6], [7], [8], [9]. As such detectors are widely used for absolute dosimetric quality assurance, those changes have been taken into account by a dedicated correction factor, $k_{\vec{B},Q}$, determined either using Monte-Carlo simulations or experimental setups [4], [5], [6], [7], [8], [9], [10]. Because these correction factors cannot be determined in clinical routine, the extent to which generic, type-specific tabulated correction factors can be applied in dosimetry protocols is an important issue. Knowing the intra-type variation, quantifying chamber-to-chamber variations within the same chamber type, enables the user to assess the applicability and uncertainty of such correction factors.

As conventional ionization chambers can lead to severe artefacts in MR-imaging, air-vented ionization chambers that produce fewer artefacts are needed for quality assurance of MR-based gating and tracking algorithms.

The MR-optimized ionization chambers studied in this paper were designed to meet these requirements. According to the vendor, the cavity dimensions, wall material, wall thickness and electrode is unchanged compared to the conventional types. In contrast to their conventional pendants, changes seem to be realized in the chamber stem to avoid artefacts in addition to production details to minimize air-layers in the chamber wall, which were observed in a recent study [9]. Due to the design changes, it is unclear if correction factors for non-MR-optimized chambers are applicable.

Thus, the aim of this work was to experimentally investigate the magnetic field correction factor $k_{\vec{B},Q}$ along with its intra-type variation and its dependencies on magnetic flux density, energy and orientation for two MR-optimized chamber types, comparing them to their conventional counterparts.

## 2 Material and methods

**Fig. 1:**
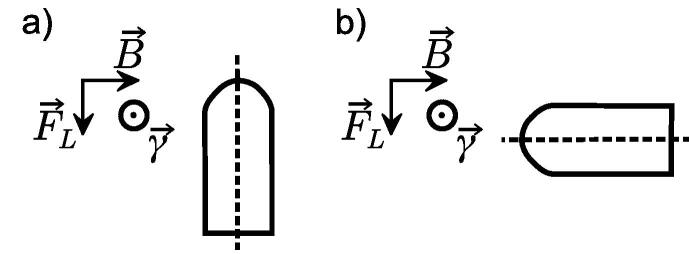
Chamber setups used in this study: a) chamber axis perpendicular to the magnetic field $\vec{B}$ and irradiation beam axis $\vec{\gamma }$. With positive *B*-values, the initial Lorentz force ${\vec{F}}_{L}$ for electrons moving along the beam axis points to the chamber stem. Negative *B*-values result in the Lorentz force pointing to the chamber tip. b) chamber axis antiparallel to the magnetic field and perpendicular to the beam axis.

Chamber readings were investigated for different magnetic flux densities *B* and beam qualities Q for four ionization chamber types: Semiflex3D-PTW31021 (SF), PinPoint3D-PTW31022 (PP), Semiflex3DMR-PTW31024 (SFMR), PinPoint3DMR-PTW31025 (PPMR) (PTW Freiburg, Germany). Measurements were carried out at Physikalisch-Technische Bundesanstalt (PTB, Germany) in a 6 x 20 x 20 cm^3^-water phantom using a mobile electromagnet (ER073W, Bruker, USA) placed in front of an Elekta Precise Linac (Elekta AB, Sweden), as specified previously [5]. The source-to-surface distance (SSD) was 110 cm and the chamber axis was positioned perpendicular to the beam axis and magnetic field lines as shown in Fig. 1a. The reference point of the chambers was positioned at 10 cm water-equivalent depth. A photon beam was collimated to 4 x 10 cm^2^ at the isocenter (SAD = 100 cm) by the MLCs. The magnetic flux density was assesed using a Hall sensor and a digital teslameter (DTM 151, Group3 Technology Limited, New Zealand). An in-house transmission monitor chamber [11] was used to monitor the accelerator’s output and normalize the detector signals. Each detector was pre-irradiated with at least 1000 MU. Measurement time was at least 80 s with constant dose rate. Each measurement signal was corrected for water temperature and atmospheric pressure. Given that only ratios of signals with and without magnetic fields are taken, polarity and recombination factors were not applied, since the literature indicates a neglectable or non-existent influence of the magnetic field on these factors [7].

### 2.1 Change of signal with change of magnetic flux density

To investigate the overall behavior of the signal of a certain chamber type in a magnetic field, the chamber reading was measured as a function of the magnetic flux density *B* at least once per chamber type using a nominal acceleration voltage of 6 MV. *B* ranged from −1.5 to 1.5 T in steps of 0.2 T, with additional steps at ± 0.35 T.

### 2.2 Chamber reading correction factor $k_{\vec{B},M,Q}$

To investigate the magnetic field effect on the chamber reading, the correction factor $k_{\vec{B},M,Q}$ was assessed by the ratio of the chamber reading at a given beam quality without ($M_{Q}$) and with an external magnetic field ($M_{Q}^{\vec{B}}$) [10].

(1) $$k_{\vec{B},M,Q}=\;\frac{M_{Q}}{M_{Q}^{\vec{B}}}$$

Magnetic flux densities representative of two MR-linacs (MRIdian, ViewRay, USA and Unity, Elekta AB, Sweden) were considered by carrying out five independent measurements for each of five chambers of each type in the 6 MV beam and magnetic flux densities of −0.35 and −1.5 T. The measurements were performed on five different days, including a full reposition of the chambers. A Mann-Whitney-U-Test was used to check for significant differences between the MR-optimized and the conventional chamber types. P-values < 0.05 were considered significant.

### 2.3 Consideration of different beam qualities

To derive $k_{\vec{B},M,Q}$ for any other beam quality than the one used in this work, we propose the quantity $c_{Q_{2}Q_{1}}$. It is the ratio of $k_{\vec{B},M,Q}$ at the desired beam quality $Q_{2}$ and the beam quality $Q_{1}$ for which $k_{\vec{B},M,Q}$ was originally determined.

(2) $$c_{Q_{2}Q_{1}}=\frac{{\left(k_{\vec{B},M,Q}\right)}_{Q_{2}}}{{\left(k_{\vec{B},M,Q}\right)}_{Q_{1}}}$$

Correction factors $k_{\vec{B},M,Q}$ were determined for 6, 10 and 15 MV with magnetic flux densities of −0.35 and −1.5 T utilizing at least two chambers per chamber type. These measurements were repeated three times per chamber on different days. $k_{\vec{B},M,Q}$ was then expressed as a function of the beam quality specifier ${\mathrm{TPR}}_{20,10}$. This specifier is described in IAEA TRS-398 [12] and was 0.683, 0.733 and 0.760 for the three nominal accelerating voltages at the accelerator used in this work [13]. A linear fit, $k_{\vec{B},M,Q}=a\cdot {\mathrm{TPR}}_{20,10}+b,$ was used to calculate $k_{\vec{B},M,Q}$ and therefore $c_{Q_{2}Q_{1}}$ for beam qualities of a 1.5 and a 0.35 T MR-linac. In this study we consider a nominal ${\mathrm{TPR}}_{20,10}$ of 0.701 for the Unity MR-linac [10] and 0.648 for the MRIdian MR-linac [14].

### 2.4 Rotation of the chamber axis

In clinical routine, cylindrical ionization chambers are generally positioned antiparallel to the magnetic field. This was not possible in our experimental setup due to the limited space between the pole shoes of the electromagnet. A 1.5 T MR-linac was used to investigate the rotational dependency of the chamber response, using the quantity $c_{\mathrm{rot}}$ as proposed by Pojtinger et al. [5].

(3) $$c_{\mathrm{rot}}=\frac{M_{{\vec{B}}_{⊥},Q}}{M_{{\vec{B}}_{\|},Q}}$$

An MR-compatible water phantom (BeamscanMR, PTW) was used. Five independent measurements of the collected charge were performed, each done with a perpendicular ($M_{{\vec{B}}_{⊥},Q}$, Fig. 1a) and an antiparallel orientation ($M_{{\vec{B}}_{\|},Q}$, Fig. 1b) of the chamber axis with respect to the magnetic field for each of two individual chambers of the two MR-optimized ionization chamber types. The chambers were positioned with their reference point in the isocenter using MV imaging. The water depth was 10 cm and the SSD 133.5 cm. The gantry angle was set to 0° and the photon beam was collimated to 10x10 cm^2^ field size at the isocenter. For every measurement, the collected charge was measured ten times with an integration time of 10 s using an electrometer (Unidos Webline, PTW). The output of the Linac was monitored using an air-vented ionization chamber (PTW31010, PTW) at a fixed position inside the radiation field.

### 2.5 Change in absorbed dose to water

To account for the change of the absorbed dose to water caused by an external magnetic field, a correction factor $c_{\vec{B}}$ can be applied [10]. To determine $c_{\vec{B}}$ for the experimental setup at PTB, a complete accelerator head model of the Elekta Precise linac was simulated with the Monte Carlo system EGSnrc [15] (Version 2021) in BEAMnrc [16], and the dose to a water voxel was determined with the egs_chamber user code presented by Wulff et al. [17].

The $c_{\vec{B}}$ values for the 1.5 T MR-linac and a 0.35 T MR-linac, 0.9936(20) and 0.9991(3), respectively, were taken from the literature [5], [10].

### 2.6 Magnetic field correction factor$k_{\vec{B},Q}$

Two mean values per chamber type were defined for the evaluation of the correction factors $k_{\vec{B},M,Q},$ $c_{Q_{2}Q_{1}}$, $c_{\mathrm{rot}}$ and $k_{\vec{B},Q}$: the arithmetic mean of a correction factor $x_{i,j}$ of repeated measurements i for a single chamber j, named ${\hat{x}}_{j},$ and the arithmetic mean of ${\hat{x}}_{j}$ for all individual chambers j, $\overset{¯}{x}$. The mathematical definition of ${\hat{x}}_{j}$, $\overset{¯}{x}$, together with the definition of their respective standard deviation SD(x) and range R(x) can be found in the supplementary material A.

As presented by van Asselen et al. [10], the change in chamber reading and change in absorbed dose to water can be used to derive ${\overset{¯}{k}}_{\vec{B},Q}$, which describes the magnetic field effects on the chamber response.

(4) $${\bar{k}}_{\vec{B},Q}={\bar{k}}_{\vec{B},M,Q}\;\cdot \;c_{\vec{B}}$$

Taking into account different beam qualities and chamber orientations by mean values of $c_{Q_{2}Q_{1}}$ and $c_{\mathrm{rot}}$, $k_{\vec{B},Q}$ for perpendicular (Eq.5) and parallel (Eq.6) orientation can be calculated as follows:

(5) $${\left({\bar{k}}_{\vec{B},Q}\right)}_{⊥,Q_{2}}={\left({\bar{k}}_{\vec{B},M,Q}\right)}_{⊥,Q_{1}}\;\cdot \;{\bar{c}}_{Q_{2}Q_{1}}\;\cdot \;{\left(c_{\vec{B}}\right)}_{Q_{2}}$$

(6) $${\left({\bar{k}}_{\vec{B},Q}\right)}_{\|,Q_{2}}={\left({\bar{k}}_{\vec{B},Q}\right)}_{⊥,Q_{2}}\;\cdot \;{\left({\bar{c}}_{\mathrm{rot}}\right)}_{Q_{2}}$$

### 2.7 Uncertainty

Uncertainties were calculated according to the Joint Committee for Guides in Metrology [18]. A detailed description is given in the supplementary material B. It was assumed that all measurement results and calculated correction factors are normally distributed. Two uncertainties were calculated for each correction factor, ${\overset{¯}{k}}_{\vec{B},M,Q}$, ${\overset{¯}{c}}_{Q_{2}Q_{1}}$, ${\overset{¯}{c}}_{\mathrm{rot}}$ and ${\overset{¯}{k}}_{\vec{B},Q}$: ${\overset{¯}{u}}_{\mathrm{ind}}$, representing the average uncertainty of all investigated individual chambers per chamber type, and ${\overset{¯}{u}}_{\mathrm{gen}}$, which additionally includes the intra-type variation as Type-B uncertainty. ${\overset{¯}{u}}_{\mathrm{ind}}$ can be compared to uncertainties in the literature, in which no intra-type variations were taken into account. ${\overset{¯}{u}}_{\mathrm{gen}}$ should be used when the generic correction factors determined in this work are applied with an (arbitrary) chamber of this type for which no more detailed knowledge is available.

## 3 Results

### 3.1 Change of signal with change of magnetic flux density

**Fig. 2:**
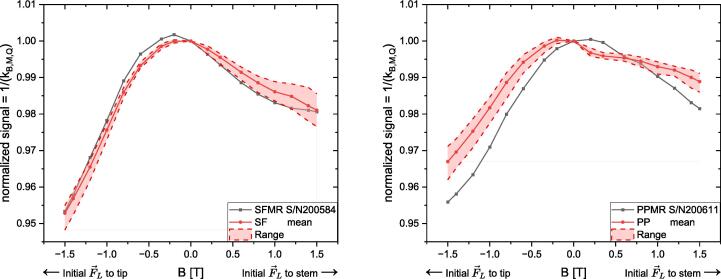
Normalized signal as a function of magnetic flux density *B* of four chamber types, two with a sensitive volume of 0.07 ccm (SF and SFMR, left) and two with a sensitive volume of 0.016 cm^3^ (PP and PPMR, right). The MR-optimized chambers were only measured once per chamber type. For the mean values of the conventional chamber types, the range band shows the maximum and minimum values of the five individual chambers.

The change of signal with change of the magnetic flux density *B* for the investigated chambers is presented in Fig. 2. In general, an increasing |*B*| led to a decrease in chamber reading and an increase in intra-type variation. The ionization chamber types with a sensitive volume of 0.07 cm^3^, SF and SFMR, showed a similar behavior, while the two ionization chamber types with a sensitive volume of 0.016  cm^3^, PP and PPMR, differed more strongly.

### 3.2 Chamber reading correction factor $k_{\vec{B},M,Q}$

**Fig. 3:**
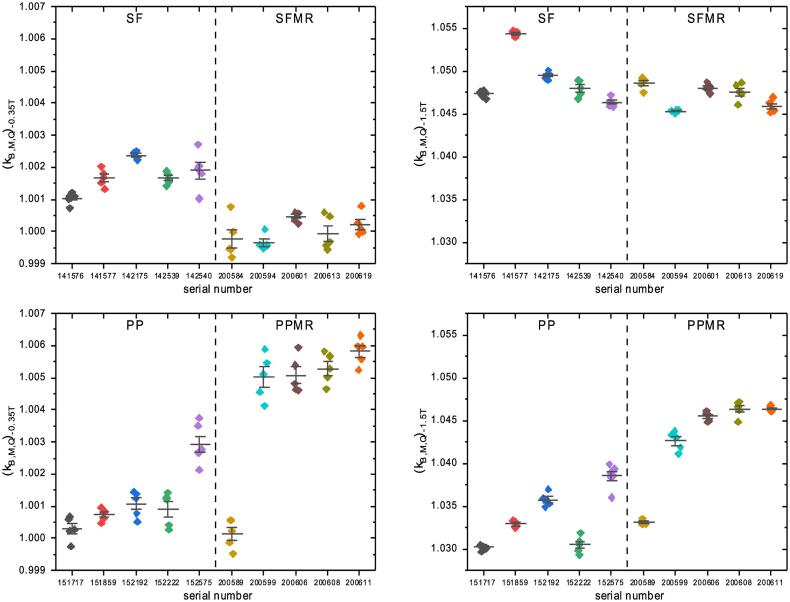
$k_{\vec{B},M,Q}$ for *B* = -0.35 T (first column) and *B* = -1.5 T (second column) as measured at a 6 MV beam on five different days, including a full reposition for five chambers of four chamber types. The MR-optimized chambers (SFMR/PPMR) are compared to their conventional counterparts (SF/PP). The horizontal line marks the mean value per individual chamber, the interval depicts the standard deviation.

$k_{\vec{B},M,Q}$ was investigated in more detail for the magnetic flux densities of –0.35 and −1.5 T. The results are presented in Fig. 3 and Table 1. For each chamber type, the mean standard deviation, $\overset{¯}{\mathrm{SD}}(k_{\vec{B},M,Q}),$ and mean range, $\overset{¯}{R}(k_{\vec{B},M,Q}),$ of reproducibility were about twice as large at –1.5 T as they were at –0.35 T. The intra-type variation, represented by $\mathrm{SD}({\hat{k}}_{\vec{B},M,Q})$ and $R\left({\hat{k}}_{\vec{B},M,Q}\right),$ was more than three times as large (Table 1).

**Table 1:** Mean, standard deviation and range of $k_{\vec{B},M,Q}$. $\overset{¯}{\mathrm{SD}}\left(k_{\vec{B},M,Q}\right)$ and $\overset{¯}{R}(k_{\vec{B},M,Q})$ represent the mean standard deviation and range over individual chambers. As such they provide information on the reproducibility of the measurements. $\mathrm{SD}({\hat{k}}_{\vec{B},M,Q})$ and $R({\hat{k}}_{\vec{B},M,Q})$ represent the standard deviation and range of all chambers of one chamber type and thus provide information about the intra-type variation. The definitions of these quantities can be found in supplementary material A.

**B = -0.35 T, Q = 0.683**	**SF**	**PP**	**SFMR**	**PPMR**
${\overset{¯}{k}}_{\vec{B},M,Q}$	1.0017	1.0012	1.0000	1.0043
$\overset{¯}{\mathrm{SD}}(k_{\vec{B},M,Q})$	0.0003	0.0004	0.0004	0.0005
$\overset{¯}{R}(k_{\vec{B},M,Q})$	0.0007	0.0010	0.0009	0.0013
$\mathrm{SD}({\hat{k}}_{\vec{B},M,Q})$	0.0005	0.0010	0.0005	0.0022
$R({\hat{k}}_{\vec{B},M,Q})$	0.0013	0.0026	0.0008	0.0057
**B = -1.5 T, Q = 0.683**				
${\overset{¯}{k}}_{\vec{B},M,Q}$	1.0491	1.0336	1.0471	1.0428
$\overset{¯}{\mathrm{SD}}(k_{\vec{B},M,Q})$	0.0005	0.0008	0.0006	0.0006
$\overset{¯}{R}(k_{\vec{B},M,Q})$	0.0013	0.0020	0.0016	0.0015
$\mathrm{SD}({\hat{k}}_{\vec{B},M,Q})$	0.0029	0.0034	0.0014	0.0052
$R({\hat{k}}_{\vec{B},M,Q})$	0.0080	0.0083	0.0033	0.0132

In case of *B* = -0.35 T, significant differences were found between SFMR and SF chambers (p = 0.01), while no significant differences were found between PPMR and PP chambers (p = 0.15). For *B* = -1.5 T, significant differences between PPMR and PP chambers were observed (p = 0.03), whereas no significant differences were seen between SFMR and SF (p = 0.42).

### 3.3 Consideration of different beam qualities

Figure S1 in the supplementary material C shows the energy dependence of ${\hat{k}}_{\vec{B}M,Q}$ for 6, 10 and 15 MV for magnetic flux densities of −0.35 and −1.5 T. All linear regressions resulted in coefficients of determination of R^2^ > 0.99 (Table S1). The correction factors ${\overset{¯}{c}}_{Q_{2}Q_{1}}$ for the transition between PTB and MR-linac beam qualities are presented in Table 2, Table 3.

**Table 2:** Summary of results: mean and standard uncertainties of different quantities $x_{i}$ for B = -0.35 T and Q = 0.683(PTB)/0.648(0.35 T MR-linac). The values of ${\overset{¯}{k}}_{\vec{B},M,Q}$, ${\overset{¯}{c}}_{Q_{2}Q_{1}}$ and ${\overset{¯}{c}}_{\mathrm{rot}}$ represent the means of these factors measured with different chambers of the same type. These mean values were used to calculate ${\overset{¯}{k}}_{\vec{B},Q}$ using equations (4), (5). The first uncertainty is ${\overset{¯}{u}}_{\mathrm{ind}}$, which represents the average uncertainty of $x_{j}$ for a single chamber, the second uncertainty is ${\overset{¯}{u}}_{\mathrm{gen}}$, which represents the uncertainty of $x_{j}$ for an arbitrary chamber using the general correction factor determined in this work and therefore includes intra-type variation. Data with * was taken from [10].

**Quantity**$x_{j}$	**SF**	**PP**	**SFMR**	**PPMR**	**Monte-Carlo simulation**
${\left({\overset{¯}{k}}_{\vec{B},M,Q}\right)}_{⊥,PTB}\pm {\overset{¯}{u}}_{\mathrm{ind}}/{\overset{¯}{u}}_{\mathrm{gen}}$	1.0017 ± 0.0021/0.0022	1.0012 ± 0.0021/0.0024	1.0000 ± 0.0021/0.0022	1.0043 ± 0.0021/0.0031	
${{(c}_{\vec{B}})}_{\mathrm{PTB}}\pm u$					0.9992 ± 0.0025
${\overset{¯}{c}}_{Q_{2}Q_{1}}\pm {\overset{¯}{u}}_{\mathrm{ind}}/{\overset{¯}{u}}_{\mathrm{gen}}$	0.9960 ± 0.0010/0.0010	0.9975 ± 0.0010/0.0010	0.9959 ± 0.0010/0.0010	0.9968 ± 0.0010/0.0011	
${{(c}_{\vec{B}})}_{0.35T\_MRL}\pm u$					*0.9991 ± 0.0003
${\left({\overset{¯}{k}}_{\vec{B},Q}\right)}_{⊥,PTB}\pm {\overset{¯}{u}}_{\mathrm{ind}}/{\overset{¯}{u}}_{\mathrm{gen}}$	1.0009 ± 0.0033/0.0033	1.0004 ± 0.0033/0.0035	0.9992 ± 0.0033/0.0033	1.0035 ± 0.0033/0.0040	
${\left({\overset{¯}{k}}_{\vec{B},Q}\right)}_{⊥,0.35T\_MRL}\pm {\overset{¯}{u}}_{\mathrm{ind}}/{\overset{¯}{u}}_{\mathrm{gen}}$	0.9968 ± 0.0029/0.0031	0.9978 ± 0.0029/0.0032	0.9950 ± 0.0029/0.0030	1.0001 ± 0.0029/0.0037

**Table 3:** Summary of results: mean and standard uncertainties of different quantities $x_{i}$ for B = -1.5 T and Q = 0.683 (PTB)/0.701(1.5 T MR-linac). The values of ${\overset{¯}{k}}_{\vec{B},M,Q}$, ${\overset{¯}{c}}_{Q_{2}Q_{1}}$ and ${\overset{¯}{c}}_{\mathrm{rot}}$ represent the means of these factors measured with different chambers of the same type. These mean values were used to calculate ${\overset{¯}{k}}_{\vec{B},Q}$ using equations (4), (5), (6). The first uncertainty is ${\overset{¯}{u}}_{\mathrm{ind}},$ which represents the average uncertainty of $x_{j}$ for a single chamber, the second uncertainty is ${\overset{¯}{u}}_{\mathrm{gen}},$ which represents the uncertainty of $x_{j}$ for an arbitrary chamber using the general correction factor determined in this work and therefore includes intra-type variation. Data with * was calculated/taken from [5].

**Quantity**$x_{j}$	**SF**	**PP**	**SFMR**	**PPMR**	**Monte-Carlo simulation**
${\left({\overset{¯}{k}}_{\vec{B},M,Q}\right)}_{⊥,PTB}\pm {\overset{¯}{u}}_{\mathrm{ind}}/{\overset{¯}{u}}_{\mathrm{gen}}$	1.0491 ± 0.0028/0.0040	1.0336 ± 0.0028/0.0044	1.0471 ± 0.0028/0.0031	1.0428 ± 0.0028/0.0059	
${{(c}_{\vec{B}})}_{\mathrm{PTB}}\pm u$					0.9967 ± 0.0025
${\overset{¯}{c}}_{Q_{2}Q_{1}}\pm {\overset{¯}{u}}_{\mathrm{ind}}/{\overset{¯}{u}}_{\mathrm{gen}}$	1.0054 ± 0.0020/0.0021	1.0048 ± 0.0020/0.0022	1.0057 ± 0.0020/0.0021	1.0063 ± 0.0020/0.0020	
${\overset{¯}{c}}_{\mathrm{rot}}\pm {\overset{¯}{u}}_{\mathrm{ind}}/{\overset{¯}{u}}_{\mathrm{gen}}$	*0.9400 ± 0.0007		0.9427 ± 0.0023/0.0025	0.9603 ± 0.0021/0.0028	
${{(c}_{\vec{B}})}_{1.5T\_MRL}\pm u$					*0.9936 ± 0.0020
${\left({\overset{¯}{k}}_{\vec{B},Q}\right)}_{⊥,PTB}\pm {\overset{¯}{u}}_{\mathrm{ind}}/{\overset{¯}{u}}_{\mathrm{gen}}$	1.0456 ± 0.0038/0.0048	1.0302 ± 0.0038/0.0051	1.0436 ± 0.0038/0.0041	1.0394 ± 0.0038/0.0064	
${\left({\overset{¯}{k}}_{\vec{B},Q}\right)}_{⊥,1.5T\_MRL}\pm {\overset{¯}{u}}_{\mathrm{ind}}/{\overset{¯}{u}}_{\mathrm{gen}}$	1.0480 ± 0.0041/0.0050	1.0319 ± 0.0040/0.0053	1.0463 ± 0.0041/0.0044	1.0426 ± 0.0040/0.0066	
${\left({\overset{¯}{k}}_{\vec{B},Q}\right)}_{\|,1.5T\_MRL}\pm {\overset{¯}{u}}_{\mathrm{ind}}/{\overset{¯}{u}}_{\mathrm{gen}}$	0.9851 ± 0.0039/0.0048		0.9863 ± 0.0045/0.0049	1.0012 ± 0.0045/0.0069	

### 3.4 Rotation of the chamber axis

Supplementary Figure S2 shows the effect of rotating the chamber axis with respect to the magnetic field of a 1.5 T MR-linac from a perpendicular to an antiparallel orientation. Both chamber types showed an increase in chamber response. In case of the SFMR, the mean increase of the response was 6.1%, resulting in${\overset{¯}{c}}_{\mathrm{rot}}$ = 0.9427 with a standard deviation of 0.0027. For the PPMR, the mean increase of the chamber response was 4.1%, making ${\overset{¯}{c}}_{\mathrm{rot}}$ equal to 0.9603 with a standard deviation of 0.0018.

### 3.5 Change in absorbed dose to water

The Monte Carlo simulation of the 6 MV PTB-setup yielded $c_{\vec{B}}$ values of 0.9992(25) and 0.9967(25), corresponding to magnetic flux densities of 0.35 and 1.5 T, respectively.

### 3.6 Magnetic field correction factor $k_{\vec{B},Q}$

Considering *B* = -1.5 T, *Q* = 0.701 and ${\overset{¯}{u}}_{\mathrm{gen}}$, we derived ${\left({\overset{¯}{k}}_{\vec{B},Q}\right)}_{\|,1.5T\_MRL}$ = 0.9863(49) in the case of the SFMR and ${\left({\overset{¯}{k}}_{\vec{B},Q}\right)}_{\|,1.5T\_MRL}$ = 1.0012(69) for a PPMR. A summary of the main results including uncertainties is presented in Table 2, Table 3.

## 4 Discussion

In this work, the characteristics of two novel MR-optimized ionization chambers, SFMR and PPMR, and their conventional counterparts, SF and PP, were investigated regarding their behavior inside an external magnetic field. Magnetic field correction factors $k_{\vec{B},Q\;}$ were derived for multiple beam qualities, magnetic flux densities *B* and chamber axis orientations. The use of several chambers of the same type further allowed the assessment of the intra-type variation.

The signals as a function of *B* determined in this work for chambers of types SF and PP were in good agreement with previously reported data by Delfs et al. and Cervantes et al. [9], [19]. The design changes made to the MR-optimized chambers had a greater impact on the PPMR type chambers than the SFMR type chambers.

With increasing *|B|*, the range of reproducibility and the intra-type variation of $k_{\vec{B},M,Q}$ increased. A similar method and the same experimental setup were employed by Pojtinger et al. [5] to examine two of the SF chambers (S/N141576 / S/N141577) also used in this work. There, $\;{\left(k_{\vec{B},M,Q}\right)}_{⊥},$ given *B* = –1.5 T, was found to differ by 0.7% between the two chambers (1.0481/1.0549). We were able to reproduce these results (1.0477/1.0545). Moreover, by using five chambers of this type, we found that one chamber (S/N141577) differed from the mean of the four other chambers, demonstrating a high intra-type variation.

In the case of the PPMR type chambers, one individual chamber exhibited a behaviour different from the other four chambers. With *B* = –1.5 T, ${\left(k_{\vec{B},M,Q}\right)}_{⊥,PTB}$ of chamber S/N200589 differed from the mean of the other chambers by 1.2%. All geometric chamber parameters were within the manufacturers tolerances according to a X-ray examination and consultation with the manufacturer. Air gaps at the chamber wall, as indicated by Cervantes et al. [9] were not observed. Also, no correlation between the calibration factor and $k_{\vec{B},M,Q}$ was found.

$c_{Q_{2}Q_{1}}$ was very uniform per chamber type and over all chamber types. Even in case of chamber SF-141577, where in the presence of a 1.5 T magnetic field ${\left(k_{\vec{B},M,Q}\right)}_{⊥,PTB}$ differed by about 0.7% from the mean of the other chambers, $c_{Q_{2}Q_{1}}$ deviated by less than 0.1% from the mean ${\overset{¯}{c}}_{Q_{2}Q_{1}}$ of this chamber type. $c_{Q_{2}Q_{1}}$ was not reported in detail by Pojtinger et al. [5], but like $\;{\left(k_{\vec{B},M,Q}\right)}_{⊥,PTB}$, it was also possible to reproduce $\;{\left(k_{\vec{B},M,Q}\right)}_{⊥,1.5T\_MRL}$ for the SF ionization chambers within 0.002 [5]. Considering the data from Pojtinger et al., the energy dependence of ${\left(k_{\vec{B},M,Q}\right)}_{⊥,PTB}\;$ for the SF chambers (S/N141576 / S/N141577) showed a linear relationship (R^2^ ≈ 0.99) of $k_{\vec{B},M,Q}$ to ${\mathrm{TPR}}_{20,10}$ over a wide range of photon energies (from 4 to 15 MV).

Pojtinger et al. [5] determined $c_{\mathrm{rot}}$ for each of two chambers of three chamber types. The maximal range of ${\overset{¯}{c}}_{\mathrm{rot}}$ per chamber type was 0.29%, the mean of all ranges was 0.17%. Compared to the maximal range and mean of range in this work (0.16%/0.13%), $c_{\mathrm{rot}}$ - like $c_{Q_{2}Q_{1}}$ – seems to have no strong correlation with $k_{\vec{B},M,Q}$ and a lower intra-type variation than $k_{\vec{B},M,Q}$. To check this assumption, we determined $c_{\mathrm{rot}}$ for the PPMR-200589, whose $k_{\vec{B},M,Q}$ factor differed most from the other chambers of this type. The chamber showed a $c_{\mathrm{rot}}$ of 0.9604 and therefore lies within the values of the other two chambers.

Given the small standard deviation and range of the correction factors ${\overset{¯}{c}}_{\mathrm{rot}}$ and ${\overset{¯}{c}}_{Q_{2}Q_{1}}$ – including chambers with high deviation of $k_{\vec{B},M,Q}$ – it can be assumed that these correction factors are constant for each chamber type. Because the relative standard deviation does not change when individual values are multiplied by a constant, the relative standard deviation determinded for ${\left({\overset{¯}{k}}_{\vec{B},M,Q}\right)}_{⊥,PTB}$ in this work can be used as a good approximation for the relative standard deviation of ${\overset{¯}{k}}_{\vec{B},Q}$. This means, that the relative uncertainty arising from intra-type variation can be estimated regardless of the positioning (perpendicular (tip) or antiparallel) and beam quality (4 to 15 MV). In addition, the relative standard deviation of the intra-type variation should not change from laboratory to laboratory.

Significant differences between the MR-optimized and the conventional chambers were found. However for *B* = -1.5 T, non-significant differences were found indicating that the correction factors determined for the SF chamber type could be used for the SFMR too. As there is no data for SFMR and PPMR published to date, $k_{\vec{B},Q}$ factors only of the SF and PP chambers could be directly compared to literature (supplementary materials Table S2). While the results of this work differ from Monte Carlo simulations presented by Margaroni et al. [8] and Cervantes et al. [20], they agree with the data of Cervantes et al. [9] and Delfs et al. [19] and the experimental data presented by Pojtinger et al. [5] and Krauss et al. [14] within the uncertainties. Discrepancies might result from the different methods and parameters used and small differences between the simulated model and experimental reality, e.g. air gap, dead volume, beam model.

In clinical practice, comparing the absorbed dose measured with the described chamber types to a reference detector may be useful. Ideally a chamber with individually determined $k_{\vec{B},Q}$ is utilized. Alternatively a Farmer-type chamber with a large sensitive volume is suitable, given its reported small intra-type variation [21], [22], [23].

In conclusion, in this study magnetic field correction factors were determined for two novel MR-optimized ionization chambers and their conventional counterparts for *B* = –0.35 T and *B* = –1.5 T, different beam qualities and chamber axis orientations. Additionally, the uncertainties of those correction factors were determined. Since for *B* = -1.5 T the intra-type variation is a non-negligible contributor to the uncertainty of a type-specific ${\overset{¯}{k}}_{\vec{B},Q}$, the intra-type variation has to be taken into account for the uncertainty of tabulated ${\overset{¯}{k}}_{\vec{B},Q}$, e.g. in future dosimetry protocols. The results of this work may help to estimate this uncertainty. Reasons for the intra-type variation and the different behaviour of MR-optimized and conventional chamber types should be investigated in the future.

## CRediT authorship contribution statement

**Stephan Frick:** Methodology, Formal analysis, Investigation, Writing – original draft, Visualization. **Moritz Schneider:** Methodology, Formal analysis, Investigation, Writing – original draft, Visualization. **Ralf-Peter Kapsch:** Conceptualization, Resources, Writing – review & editing, Supervision. **Daniela Thorwarth:** Conceptualization, Resources, Writing – review & editing, Supervision.

## Declaration of Competing Interest

The authors declare the following financial interests/personal relationships which may be considered as potential competing interests: The Department of Radiation Oncology Tübingen receives financial and technical support by Elekta, Philips, Siemens, Dr. Sennewald Medizintechnik, Kaiku Health, TheraPanacea, PTW and ITV in the context of research cooperations.

## References

[1] Keall P.J., Brighi C., Glide-Hurst C., Liney G., Liu P.Z.Y., Lydiard S. (2022). Integrated MRI-guided radiotherapy — opportunities and challenges. Nat Rev Clin Oncol.

[2] Winkel D., Bol G.H., Kroon P.S., van Asselen B., Hackett S.S., Werensteijn-Honingh A.M. (2019). Adaptive radiotherapy: the Elekta Unity MR-linac concept. Clin Transl Radiat Oncol.

[3] Akdag O., Borman P.T.S., Woodhead P., Uijtewaal P., Mandija S., Van Asselen B. (2022). First experimental exploration of real-time cardiorespiratory motion management for future stereotactic arrhythmia radioablation treatments on the MR-linac. Phys Med Biol.

[4] Meijsing I., Raaymakers B.W., Raaijmakers A.J.E., Kok J.G.M., Hogeweg L., Liu B. (2009). Dosimetry for the MRI accelerator: the impact of a magnetic field on the response of a farmer NE2571 ionization chamber. Phys Med Biol.

[5] Pojtinger S., Nachbar M., Ghandour S., Pisaturo O., Pachoud M., Kapsch R.-P. (2020). Experimental determination of magnetic field correction factors for ionization chambers in parallel and perpendicular orientations. Phys Med Biol.

[6] Spindeldreier C.K., Schrenk O., Bakenecker A., Kawrakow I., Burigo L., Karger C.P. (2017). Radiation dosimetry in magnetic fields with farmer-type ionization chambers: determination of magnetic field correction factors for different magnetic field strengths and field orientations. Phys Med Biol.

[7] de Prez L., Woodings S., de Pooter J., van Asselen B., Wolthaus J., Jansen B. (2019). Direct measurement of ion chamber correction factors, k Q and k B, in a 7 MV MRI-linac. Phys Med Biol.

[8] Margaroni V., Pappas E.P., Episkopakis A., Pantelis E., Papagiannis P., Marinos N. (2023). Dosimetry in 1.5 T MR-linacs: Monte Carlo determination of magnetic field correction factors and investigation of the air gap effect. Med Phys.

[9] Cervantes Y., Billas I., Shipley D., Duane S., Bouchard H. (2020). Small-cavity chamber dose response in megavoltage photon beams coupled to magnetic fields. Phys Med Biol.

[10] van Asselen B., Woodings S.J., Hackett S.L., van Soest T.L., Kok J.G.M., Raaymakers B.W. (2018). A formalism for reference dosimetry in photon beams in the presence of a magnetic field. Phys Med Biol.

[11] Kapsch R-P, Krauss A. On the performance of monitor chambers to measure the output of medical linear accelerators for high-precision dosimetric investigations. In: Dössel O, Schlegel WC, editors. World Congr. Med. Phys. Biomed. Eng. Sept. 7 - 12 2009 Munich Ger., vol. 25/1, Berlin, Heidelberg: Springer Berlin Heidelberg; 2009, p. 85–8. https://doi.org/10.1007/978-3-642-03474-9_25.

[12] IAEA TRS 398: Absorbed Dose Determination in External Beam Radiotherapy: An International Code of Practice for Dosimetry based on Standards of Absorbed Dose to Water n.d.

[13] Krauss A., Kapsch R.P. (2014). Experimental determination of *k _Q_* factors for cylindrical ionization chambers in 10 cm × 10 cm and 3 cm × 3 cm photon beams from 4 MV to 25 MV. Phys Med Biol.

[14] Krauss A., Spindeldreier C.K., Klüter S. (2020). Direct determination of kB, Q, Q0 for cylindrical ionization chambers in a 6 MV 0.35 T MR-linac. Phys Med Biol.

[15] Kawrakow I. (2001). The EGSnrc code system, Monte Carlo simulation of electron and photon transport. NRCC Rep Pirs-701.

[16] Rogers D., Faddegon B., Ding G., Ma C., We J., Mackie T. (1995). BEAM: a Monte Carlo code to simulate radiotherapy treatment units. Med Phys.

[17] Wulff J., Zink K., Kawrakow I. (2008). Efficiency improvements for ion chamber calculations in high energy photon beams: efficiency improvements for ion chamber calculations. Med Phys.

[18] Guide to the expression of uncertainty in measurement (GUM). Int Organ Stand Geneva 1995; ISBN 92-67-10188-9.

[19] Delfs B., Blum I., Tekin T., Schönfeld A., Kranzer R., Poppinga D. (2021). The role of the construction and sensitive volume of compact ionization chambers on the magnetic field-dependent dose response. Med Phys.

[20] Cervantes Y., Duchaine J., Billas I., Duane S., Bouchard H. (2021). Monte Carlo calculation of detector perturbation and quality correction factors in a 1.5 T magnetic resonance guided radiation therapy small photon beams. Phys Med Biol.

[21] Smit K., Van Asselen B., Kok J.G.M., Aalbers A.H.L., Lagendijk J.J.W., Raaymakers B.W. (2013). Towards reference dosimetry for the MR-linac: magnetic field correction of the ionization chamber reading. Phys Med Biol.

[22] Woodings S.J., Asselen B., Soest T.L., Prez L.A., Lagendijk J.J.W., Raaymakers B.W. (2019). Technical note: consistency of PTW30013 and FC65-G ion chamber magnetic field correction factors. Med Phys.

[23] Alissa M., Zink K., Kapsch R., Schoenfeld A.A., Frick S., Czarnecki D. (2023). Experimental and Monte Carlo-based determination of magnetic field correction factors kB, Q in high-energy photon fields for two ionization chambers. Med Phys.

