# Supplementary material for: Experimental characterization of four ionization chamber types in magnetic fields including intra-type variation
Source: Phys Imaging Radiat Oncol. 2024 Feb 25;29:100561. doi: 10.1016/j.phro.2024.100561 (PMC10924196; doi:10.1016/j.phro.2024.100561)
Supplement: Supplementary data 1 [file mmc1.docx]

# Supplementary materials

## *A* *Definitions for the evaluation of reproducibility and intra-type variation*

The arithmetic mean of a correction factor $x_{i,j}$of $n$ repeated measurements $i$ of a single chamber $j$, $\hat{x}_{j}$*,* and the arithmetic mean for $N$ individual chambers $j$, $\bar{x}$, are defined as:

$$\hat{x}_{j}= \frac{1}{n}\sum_{i=1}^{n} x_{i,j}$$

$$\bar{x}=\frac{1}{N}\sum_{j=1}^{N} \hat{x}_{j}= \frac{1}{n*N} \sum_{j=1}^{N} \sum_{i=1}^{n} x_{i,j}$$

Standard deviation *SD* of reproducibility of one chamber:

$$SD\left( x_{j} \right)= \sqrt{\frac{1}{n-1}\sum_{i=1}^{n} {(x_{i,j}-\hat{x}_{j})}^{2}}$$

Mean standard deviation of reproducibility of one chamber type:

$$\bar{SD}\left( x_{j} \right)= \frac{1}{N}\sum_{j=1}^{N} SD\left( x_{j} \right)$$

Range *R* of reproducibility of one chamber:

$$R\left( x_{j} \right)=Range \left\{ x_{i=1,j},\ldots,x_{i=n,j} \right\}=max \left\{ x_{i=1,j},\ldots,x_{i=n,j} \right\}-min \{x_{i=1,j},\ldots,x_{i=n,j}\}$$

Mean range of reproducibility of one chamber type:

$$\bar{R}\left( x_{j} \right)=\frac{1}{N}\sum_{j=1}^{N} R\left( x_{j} \right)$$

Standard deviation of intra-type variation of one chamber type:

$$SD(\hat{x})=\sqrt{\frac{1}{N-1}\sum_{j=1}^{N} {(\hat{x}}_{j}-\bar{x})^{2}}$$

Mean range of intra-type variation of one chamber type:

$$R(\hat{x})=Range\{\hat{x}_{j=1},\ldots,\hat{x}_{j=N}\}$$

## *B Calculation of the Uncertainty*

The uncertainty was calculated according to the following steps:

1. Type-A uncertainties for $\bar{k}_{\vec{B},M,Q}$, $\bar{c}_{Q_{2}Q_{1}}$ and $\bar{c}_{rot}$ were estimated using the standard error of the mean of uncorrelated repeated measurements with the same chamber $j$, $u_{A,j}= \frac{SD\left( x_{j} \right)}{\sqrt{n}}$*,* where *n* is the number of repeated measurements. Since these factors were measured with more than one chamber per chamber type, the arithmetic mean of this uncertainty was calculated from all $N$ chambers of the chamber type used, i.e. $\frac{1}{N}\sum_{j=1}^{N} u_{A,j}$.
2. The Type-B uncertainty at PTB for $\bar{k}_{\vec{B},M,Q}$ with 6 MV was estimated to 0.21% and 0.27% for B = ‑0.35 T and ‑1.5 T, respectively, taking into account uncertainties for temperature and atmospheric pressure measurements (calculated on the basis of information from the instrument manufacturer) as well as additional uncertainties for positioning, the electrometer and the influence of the magnetic field on the signal from the external monitor chamber.
3. The Type-B uncertainty at PTB for $\bar{c}_{Q_{2}Q_{1}}$ was estimated to 0.1% and 0.2% for B = ‑0.35 T and ‑1.5 T, respectively by taking into account uncertainties for ${TPR}_{20,10}$, plus an additional uncertainty for the small number of chambers examined.
4. The Type-B uncertainty for the 1.5 T MR-linac for $\bar{c}_{rot}$ was estimated to 0.2% by considering uncertainties for positioning, temperature and atmospheric pressure measurements and the leakage current.
5. For $\bar{u}_{gen}$, an additional Type-B uncertainty for $\bar{k}_{\vec{B},M,Q}$, $\bar{c}_{Q_{2}Q_{1}}$ and $\bar{c}_{rot}$ was estimated by taking into account the intra-type variation. This uncertainty corresponds to $SD(\hat{x})$.
6. The uncertainty at PTB for $c_{\vec{B}}$ includes the Monte Carlo variance as well as an additional uncertainty of 0.2%, taking account of the possibility of systematic Monte Carlo uncertainties [1]. The uncertainty as well as the value of $c_{\vec{B}}$ for the MR-linacs were taken from literature [2,3].
7. For the final uncertainty of $\bar{k}_{\vec{B},M,Q}$, $\bar{c}_{Q_{2}Q_{1}}$, $\bar{c}_{rot}$ and $c_{\vec{B}}$, the square root of the squared sum of Type-A and Type-B uncertainties was calculated. The uncertainty $\bar{u}_{gen}$ differs from $\bar{u}_{ind}$ by taking v. into account.
8. For the uncertainty of the final correction factor $\bar{k}_{\vec{B},Q}$, the combined standard uncertainty was calculated with equation 7. The function *f* is described in formular 4, 5 and 6 – depending on which ${u(\bar{k}}_{\vec{B},Q})$ is calculated.

$u_{c}^{2}\left( y \right)=\sum_{i=1}^{N} \left( \frac{\partial f}{\partial x_{i}} \right)^{2} u^{2} (x_{i})$ (7)

## *C Beam quality dependence*

**Table S.1** Parameters for the linear fit $k_{\vec{B},M,Q}\left( {TPR}_{20,10} \right)= a\cdot({TPR}_{20,10}) +b$ for B = -0.35 T and B = -1.5 T. The first number is the mean value of at least two chambers, the second number is the standard uncertainty estimation (rectangle distribution) with $u= \frac{range}{\sqrt{3}}$.

| *B* = -0.35 T | SF | PP | SFMR | PPMR |
| --- | --- | --- | --- | --- |
| Mean a (slope) | 0.1158 ± 0.0037 | 0.0700 ± 0.0042 | 0.1206 ± 0.0016 | 0.0924 ± 0.0044 |
| Mean b | 0.9224 ± 0.0028 | 0.9536 ± 0.0036 | 0.9178 ± 0.0016 | 0.9424 ± 0.0029 |
| Mean R^2^ | 0.9985 ± 0.0020 | 0.9956 ± 0.0029 | 0.9966 ± 0.0018 | 0.9986 ± 0.0015 |
| *B* = -1.5 T |  |  |  |  |
| Mean a (slope) | 0.3336 ± 0.0311 | 0.2985 ± 0.0328 | 0.3347 ± 0.0148 | 0.3804 ± 0.0025 |
| Mean b | 0.8221 ± 0.0259 | 0.8308 ± 0.0271 | 0.8186 ± 0.0112 | 0.7861 ± 0.0016 |
| Mean R^2^ | 0.9969 ± 0.0030 | 0.9926 ± 0.0059 | 0.9996 ± 0.0003 | 0.9974 ± 0.0009 |

**Figure S.1** Energy dependence of $k_{\vec{B},M,Q}$ with *B* = -0.35 T (first column) and *B* = -1.5 T (second column) for the four investigated chamber types. Each point represents the average of three independent measurements. The intervals indicate the standard deviation. Note the different vertical axis scales used in the left- and right-hand columns.

## *D Influence of chamber orientation*

**Figure S.2** Correction factor c_rot_ to account for the rotation of the chamber axis with respect to the magnetic field from a perpendicular to an antiparallel orientation in a 1.5 T MR-linac for ionization chambers of types SFMR (left, grey) and PPMR (right, red).

## *E Comparison of results with literature*

| **literature** | **SF** | **u** | **PP** | **u** | **orientation** | **energy [MV]** | **B [T]** |
| --- | --- | --- | --- | --- | --- | --- | --- |
| This study | 0.9851 | 0.0048 |  |  | anti-parallel | 7 | 1.5 |
| Margaroni2023 [4] | 0.9982 | 0.0044 | 0.9962 | 0.0044 | anti-parallel | 7 | 1.5 |
| Pojtinger2020 [2] | 0.9841 | 0.0030 |  |  | anti-parallel | 7 | 1.5 |
| Margaroni2023 [4] | 1.0019 | 0.0043 | 1.0022 | 0.0044 | parallel | 7 | 1.5 |
| Cervantes2021 [5] | 1.0160 | 0.0040 | 0.9970 | 0.0040 | parallel | 7 | 1.5 |
| This study | 1.0480 | 0.0050 | 1.0319 | 0.0044 | perpendicular (tip) | 7 | 1.5 |
| Margaroni2023 [4] | 1.0167 | 0.0043 | 1.0127 | 0.0045 | perpendicular (tip) | 7 | 1.5 |
| Cervantes2021 [5] | 0.9720 | 0.0040 | 0.9940 | 0.0044 | perpendicular (tip) | 7 | 1.5 |
| Pojtinger2020 [2] | 1.0478 | 0.0030 |  |  | perpendicular (tip) | 7 | 1.5 |
| This study | 1.0456 | 0.0048 | 1.0302 | 0.0051 | perpendicular (tip) | 6 | 1.5 |
| Cervantes2020 [6] | 1.0399 | 0.0036 | 1.0243 | 0.0058 | perpendicular (tip) | 6 | 1.5 |
| Delfs2021 [7] | 1.0400 | 0.0057 | 1.0375 | 0.0057 | perpendicular (tip) | 6 | 1.5 |
| This study | 1.0009 | 0.0033 | 1.0004 | 0.0035 | perpendicular (tip) | 6 | 0.35 |
| Delfs2021 [7] | 1.0000 | 0.0057 | 1.0000 | 0.0057 | perpendicular (tip) | 6 | 0.35 |
| Cervantes2020 [6] | 1.0062 | 0.0036 | 1.0107 | 0.0056 | perpendicular (tip) | 6 | 0.35 |
| This study | 0.9968 | 0.0031 | 0.9978 | 0.0032 | perpendicular (tip) | 6 (0.35T-MRL) | 0.35 |

**Table S.2:** Comparison of results of $k_{\vec{B},Q}$ from this study with the literature. For the comparison of uncertainties, $\bar{u}_{ind}$ is given for the results of this study.

**References**

[1] Wulff J, Heverhagen JT, Zink K, Kawrakow I. Investigation of systematic uncertainties in Monte Carlo-calculated beam quality correction factors. Phys Med Biol 2010;55:4481–93. https://doi.org/10.1088/0031-9155/55/16/S04.

[2] Pojtinger S, Nachbar M, Ghandour S, Pisaturo O, Pachoud M, Kapsch R-P, et al. Experimental determination of magnetic field correction factors for ionization chambers in parallel and perpendicular orientations. Phys Med Biol 2020;65:245044. https://doi.org/10.1088/1361-6560/abca06.

[3] van Asselen B, Woodings SJ, Hackett SL, van Soest TL, Kok JGM, Raaymakers BW, et al. A formalism for reference dosimetry in photon beams in the presence of a magnetic field. Phys Med Biol 2018;63:125008. https://doi.org/10.1088/1361-6560/aac70e.

[4] Margaroni V, Pappas EP, Episkopakis A, Pantelis E, Papagiannis P, Marinos N, et al. Dosimetry in 1.5 T MR‐Linacs: Monte Carlo determination of magnetic field correction factors and investigation of the air gap effect. Med Phys 2023;50:1132–48. https://doi.org/10.1002/mp.16082.

[5] Cervantes Y, Duchaine J, Billas I, Duane S, Bouchard H. Monte Carlo calculation of detector perturbation and quality correction factors in a 1.5 T magnetic resonance guided radiation therapy small photon beams. Phys Med Biol 2021;66:225004. https://doi.org/10.1088/1361-6560/ac3344.

[6] Cervantes Y, Billas I, Shipley D, Duane S, Bouchard H. Small-cavity chamber dose response in megavoltage photon beams coupled to magnetic fields. Phys Med Biol 2020;65:245008. https://doi.org/10.1088/1361-6560/aba6d6.

[7] Delfs B, Blum I, Tekin T, Schönfeld A, Kranzer R, Poppinga D, et al. The role of the construction and sensitive volume of compact ionization chambers on the magnetic field‐dependent dose response. Med Phys 2021;48:4572–85. https://doi.org/10.1002/mp.14994.
